# Approaches to modeling cancer metastasis: from bench to bedside

**DOI:** 10.3389/fonc.2025.1602489

**Published:** 2025-08-08

**Authors:** Leiyu Sun, Yajuan Zhou, Maoxu Yin, Fei Wang, Lijuan Yang

**Affiliations:** ^1^ Department of Gastroenterology, Binzhou Medical University Hospital, Binzhou, Shandong, China; ^2^ Medical Research Center, Binzhou Medical University Hospital, Binzhou, China

**Keywords:** cancer metastasis, metastasis models, preclinical research, patient-derived models (PDX, organoids), therapeutic evaluation, metastasis mechanisms

## Abstract

Cancer metastasis modeling requires multidisciplinary approaches that integrate experimental, computational, and clinical research to unravel the complexities of cancer spread). By deepening our understanding of the metastatic process, researchers can efficiently and precisely develop targeted therapies and personalized treatment strategies to relieve the burden of metastasis on patients. In this review, we highlight the critical roles of experimental models in advancing knowledge of metastasis, identifying therapeutic targets, evaluating treatment strategies, and improving patient outcomes. These models serve as essential tools for translational research and drug discovery in the fight against metastatic cancer

## Introduction

**Figure 1:**
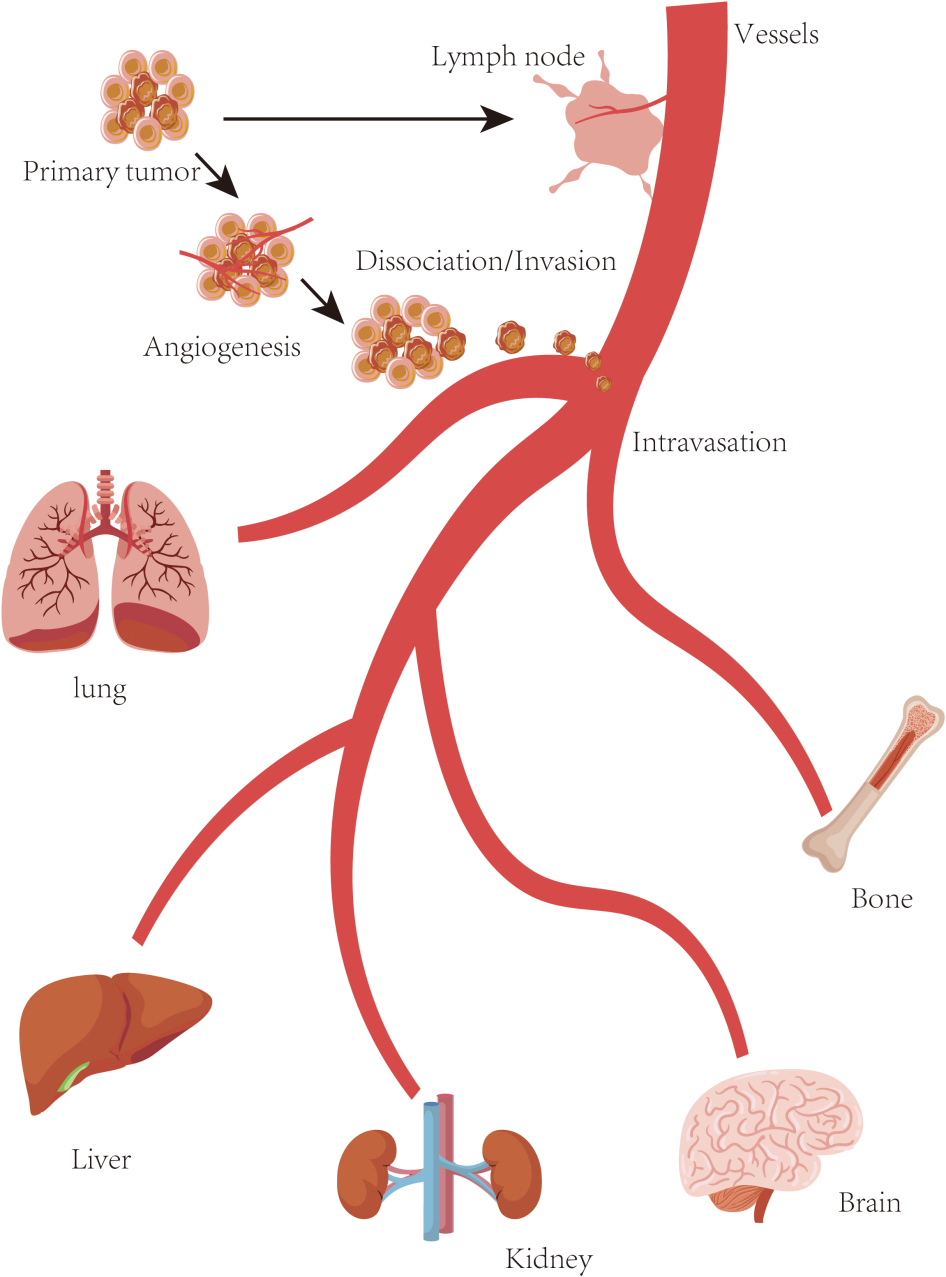
Metastasis. Schematic overview of the metastatic cascade. The multistep process of cancer metastasis includes local invasion, intravasation into blood or lymphatic vessels, survival in circulation, extravasation at distant sites, and colonization of secondary organs (lung, liver, kidney, brain, bone, et al.).

Cancer metastasis is a complex, multistep process in which cancer cells spread from the primary tumor to distant sites throughout the body (1, 2) (**Figure 1**). Understanding the mechanisms underlying metastasis is essential for developing effective therapeutic strategies. To this end, a wide range of experimental models—including xenograft models, orthotopic models, genetically engineered mouse models (GEMMs), and *in vivo* imaging approaches—have been established to investigate different aspects of metastatic progression (3). Each model offers unique advantages and limitations, and selecting the appropriate model depends on the specific research questions and objectives (4). Importantly, integrating multiple experimental models can yield a more comprehensive understanding of metastasis and enhance the translational relevance of preclinical findings. This review provides an overview of the existing, widely used, and emerging models of cancer metastasis for basic, translational, and clinical research. We highlight their critical roles in elucidating metastatic mechanisms, advancing metastasis-specific therapeutic strategies, and establishing clinically relevant platforms for drug development and therapeutic evaluation in patients with metastatic cancer.

Modeling cancer metastasis plays a pivotal role in advancing our understanding of the metastatic process and its clinical implications. Experimental metastasis models are essential for identifying metastasis-related genes and pathways, assessing therapeutic strategies, investigating tumor–microenvironment interactions, evaluating imaging techniques, enabling personalized medicine, discovering biomarkers, and conducting preclinical testing for drug development (5). These models allow researchers to dissect the molecular and cellular mechanisms driving metastasis by recapitulating key steps of the process, including invasion, intravasation, circulation, extravasation, and colonization. By comparing metastatic and non-metastatic cells or tissues, researchers can identify genetic and epigenetic alterations that promote metastatic progression, providing valuable insights into potential therapeutic targets (6). Experimental models are also indispensable for evaluating the efficacy of anti-metastatic therapies. Preclinical testing (7) of drugs or combination treatments in these models helps determine their ability to inhibit metastasis, reduce tumor burden at distant sites, and improve overall survival, serving as a critical step before advancing to clinical trials. Since the tumor microenvironment profoundly influences metastasis, these models enable researchers to investigate interactions between cancer cells and surrounding stromal cells, immune cells, extracellular matrix components, and vasculature. Such interactions regulate processes like epithelial-to-mesenchymal transition (EMT) (8), angiogenesis, immune evasion, and metastatic colonization.

Metastasis models are also essential for developing and optimizing imaging techniques to detect and monitor metastatic lesions. The use of fluorescent probes, radiolabeled tracers, and advanced imaging modalities facilitates real-time visualization of cancer cell dissemination, assessment of metastatic potential, monitoring of treatment response, and evaluation of imaging-guided therapeutic interventions (9). Patient-derived experimental models, such as xenografts and organoids, enable the study of metastasis in the context of individual tumors. These models provide a platform for identifying predictive biomarkers, evaluating personalized treatment responses, and informing clinical decision-making (10). Overall, metastasis research models serve as a crucial bridge between basic research and clinical translation. Extensive exploration and refinement of these models will greatly enhance our understanding of metastatic mechanisms, aid in the identification of therapeutic targets, improve treatment strategies, and ultimately contribute to better outcomes for patients with metastatic cancer.

## Cancer metastasis modeling history

Cancer metastasis modeling has evolved as our understanding of the disease has advanced. The phenomenon of cancer metastasis was first documented in the 19th century, but the underlying mechanisms were not well defined at the time (11). Surgeons noticed that some patients developed tumors in distant sites after surgical removal of the primary tumor implying that cancer could spread throughout the body.

In the early 20th century, scientists found that cancer cells can grow and form tumors in different hosts by transplanting tumor fragments or cells from one animal to another, which laid the foundation for studying tumor biology and provided early insights into the metastatic potential of cancer cells. In the mid-20th century, researchers began developing experimental models to focus on the spread of cancer. This research involved injecting cancer cells into experimental animals, primarily mice, to investigate their ability to form secondary tumors at distant sites in which these models allowed for controlled experimentation and provided a framework to study the different steps of metastasis. The adventure of cell culture techniques, such as establishing cancer cell lines, allowed researchers to study cancer cells in a controlled laboratory setting since the late 20^th^ century and facilitated the investigation of cellular and molecular mechanisms underlying metastasis, including cell migration, invasion, and adhesion (12).

Cancer research was further revolutionized by the advent of genetic engineering techniques in the late 20th century. Genetically engineered mouse models (GEMMs) that carried specific genetic alterations found in human cancers were established. These models allowed for the study of tumor initiation, progression, and metastasis in a more physiologically relevant context. In the meantime, xenograft models, where human tumor cells are transplanted into immunocompromised mice, became widely used for studying cancer metastasis. This approach allowed for the transplantation of human cancer cells and the assessment of their metastatic potential in an *in vivo* system (13).

Imaging technologies have gained substantial advancements since the beginning of the 21st century especially in intravital microscopy and bioluminescence imaging (14). These techniques enabled real-time visualization and tracking of cancer cells in live animals to provide dynamic insights into the metastatic process (15).

Patient-derived models, such as patient-derived xenografts (PDX) and organoids, have been introduced more recently for studying cancer metastasis. These models better preserve the genetic and molecular heterogeneity of human tumors and allow for personalized medicine approaches and drug testing (16).

In summary, the history of cancer metastasis modeling is outlined by a progression from observational studies to the development of controlled experimental models that mimic different aspects of metastasis (17). All those models have played a crucial role in advancing our understanding of cancer metastasis and have facilitated the development of new therapies and treatment strategies (18).

## Non-mammalian cancer metastasis models

**Figure 2:**
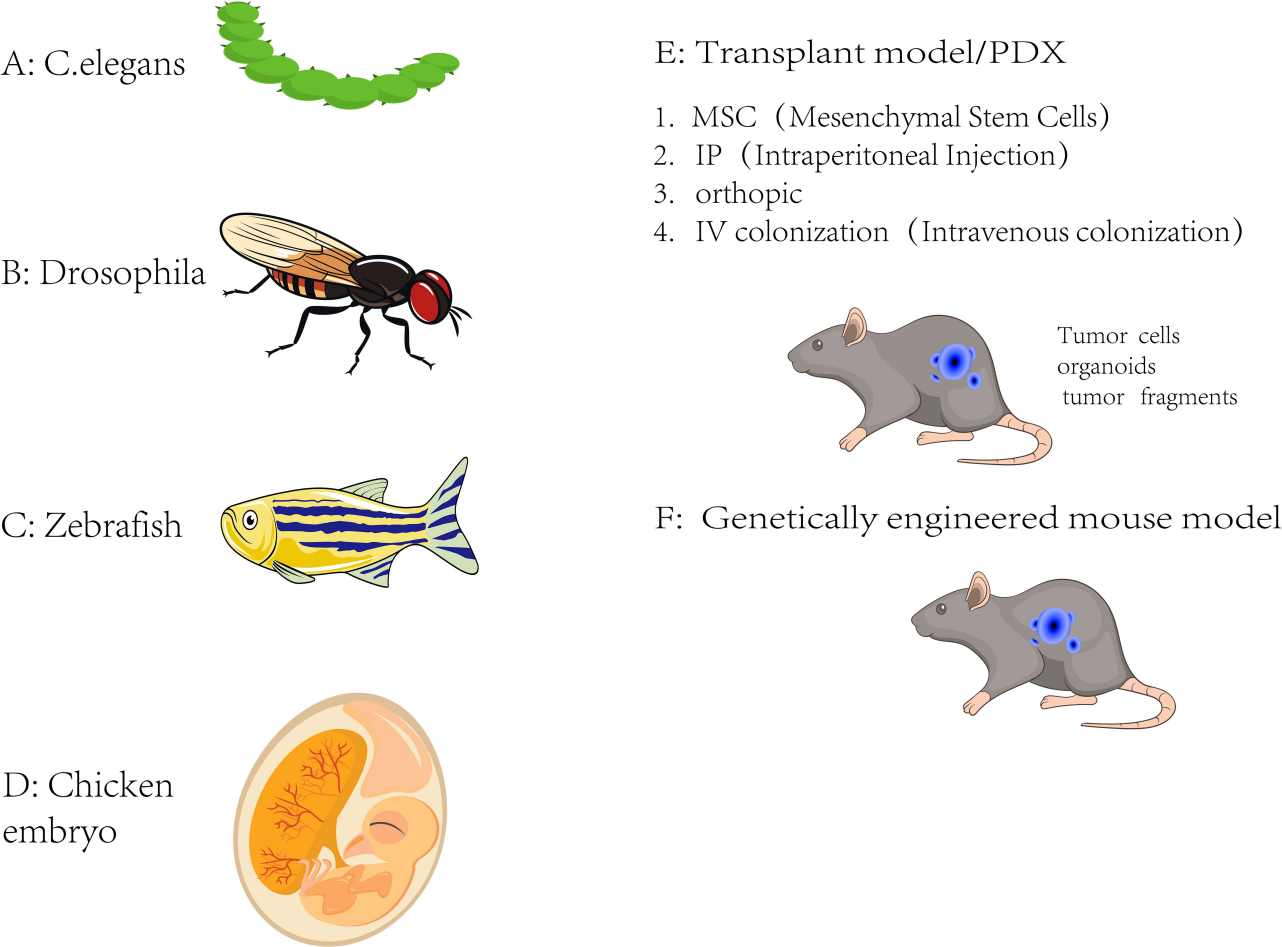
Metastasis model. Currently available *in vivo* metastasis models. **(A)** C. elegans, **(B)** Drosophila melanogaster, **(C)** Zebrafish, **(D)** Chicken embryo, **(E)** Transplantation-based models (including PDX), involving mesenchymal stem cells (MSC), intraperitoneal injection (IP), orthotopic transplantation, and intravenous colonization (IV), **(F)** Genetically engineered mouse models (GEMMs), which simulate spontaneous tumor initiation and metastasis in vivo.

While mammalian models like mice are the most adapted models for studying cancer metastasis, non-mammalian models utilized to gain insights into metastatic processes are also available. These non-mammalian models offer more benefits such as lower cost, ease of manipulation, and unique experimental opportunities (19). Some non-mammalian models such as zebrafish (Danio rerio), drosophila melanogaster (Fruit Fly), caenorhabditis elegans (Nematode), chick embryo chorioallantoic membrane (CAM) assay, and cnidarians (Hydra and Sea Anemones) are currently selected in cancer metastasis research (**Figure 2**).

Zebrafish (Danio rerio) has been a powerful model for studying cancer metastasis, in which its transparent embryos allow direct visualization of cancer cell behavior and interactions with host tissues (20). The genetic and molecular similarities between zebrafish and humans make this model valuable in studying cancer cell migration, invasion, extravasation, and colonization for understanding metastasis and screening anti-metastatic drugs (21).

Fruit flies (Drosophila melanogaster) can be easily manipulated with well-characterized genetics to study various biological processes, including cancer metastasis (22). Drosophila models were frequently employed to investigate cellular invasion, epithelial-mesenchymal transition (EMT), and interactions between cancer cells and the tumor microenvironment (23) in identifying novel metastasis-regulating genes and signaling pathways.

As a transparent roundworm, C. elegans (Caenorhabditis elegans) are widely applied to study various biological processes (24). Despite lacking an immune system, its simple anatomy and well-defined cell lineage allow for precise observations of cancer cell behavior by using C. elegans to explore aspects of metastasis such as cell migration, invasion, and response to therapeutic interventions.

Chick embryo chorioallantoic membrane (CAM) assay involves grafting tumor cells onto the chick embryo CAM as an ex vivo model which provides a rich vascular network, facilitating tumor cell growth and angiogenesis (25). This assay has been used to study tumor cell invasion, angiogenesis, and the effects of anti-angiogenic therapies. It is required for assessing tumor cell interactions with the vasculature during metastasis.

Cnidarians (Hydra and Sea Anemones) are simple aquatic organisms exhibiting regenerative capabilities. Some species of hydra and sea anemones have been used as models for studying cancer cell migration, invasion, and regeneration (26). These models offer the advantage of easy manipulation and can provide insights into the early evolutionary origins of metastatic processes.

While non-mammalian models provide valuable insights into metastatic processes, they have limitations. They may not fully recapitulate the complexity of human metastasis, including interactions with the immune system and organ-specific microenvironments. Therefore, non-mammalian models are often used complementary to mammalian models to provide additional insights and novel perspectives in cancer metastasis research (27).

## Mammalian cancer metastasis models

Mammalian cancer metastasis models are experimental systems used to study the process of cancer metastasis, which is the spread of cancer cells from the primary tumor to distant organs or tissues in the body. These models are designed to replicate the complex interactions and steps involved in metastasis, allowing researchers to investigate the underlying mechanisms and test potential therapeutic interventions (27). Here are some mammalian cancer metastasis models commonly used in research discussed next.To better understand the strengths and limitations of currently available metastasis models, **Table 1** summarizes the key characteristics of several commonly used experimental systems (**Table 1**).

**Table 1:** Comparison of common experimental models of metastasis.

Model type	Species	Modeling approach	Immune system status	Metastasis simulation	Timeframe	Cost	Advantages	Limitations
Subcutaneous Xenograft	Mouse (nude/NSG)	Subcutaneous injection	Immunodeficient	No	Fast	Low	Easy to perform; good for tumor growth assessment	Does not mimic metastasis or tumor microenvironment
Orthotopic Xenograft	Mouse	Injection into organ of origin	Immunodeficient or humanized	Yes	Moderate	Moderate	Better mimics tumor microenvironment; allows metastasis	Technically demanding; limited immune system interactions
Zebrafish Xenograft	Zebrafish embryo	Yolk sac or vessel injection	Immunodeficient (larval stage)	Yes	Very fast	Very low	Transparent body allows real-time imaging; high-throughput	Evolutionary distance; limited immune relevance
Genetically Engineered Mouse Model (GEMM)	Mouse	Spontaneous tumor formation via genetic modification	Immunocompetent	Yes	Slow	High	Natural tumor progression; intact immune system	Long development time; costly; technically complex
Patient-Derived Organoid with Metastatic Assay (PDO-based)	Human/Mouse (in vivo validation)	Organoid culture ± injection	Partial (if co-cultured)	Variable	Moderate	Moderate	Maintains patient-specific heterogeneity; useful for drug testing	Limited metastatic niche simulation; lacks systemic interaction

## PDX-based cancer metastasis models

PDX-based cancer metastasis models, also known as patient-derived xenograft models, are preclinical models that involve the implantation of patient-derived tumor tissue directly into immunocompromised mice or other animal hosts. These models are developed to preserve the genetic and phenotypic characteristics of the original tumor, including its metastatic potential, allowing researchers to study the metastatic behavior of cancer cells in a more clinically relevant context (28).

**Figure 3:**
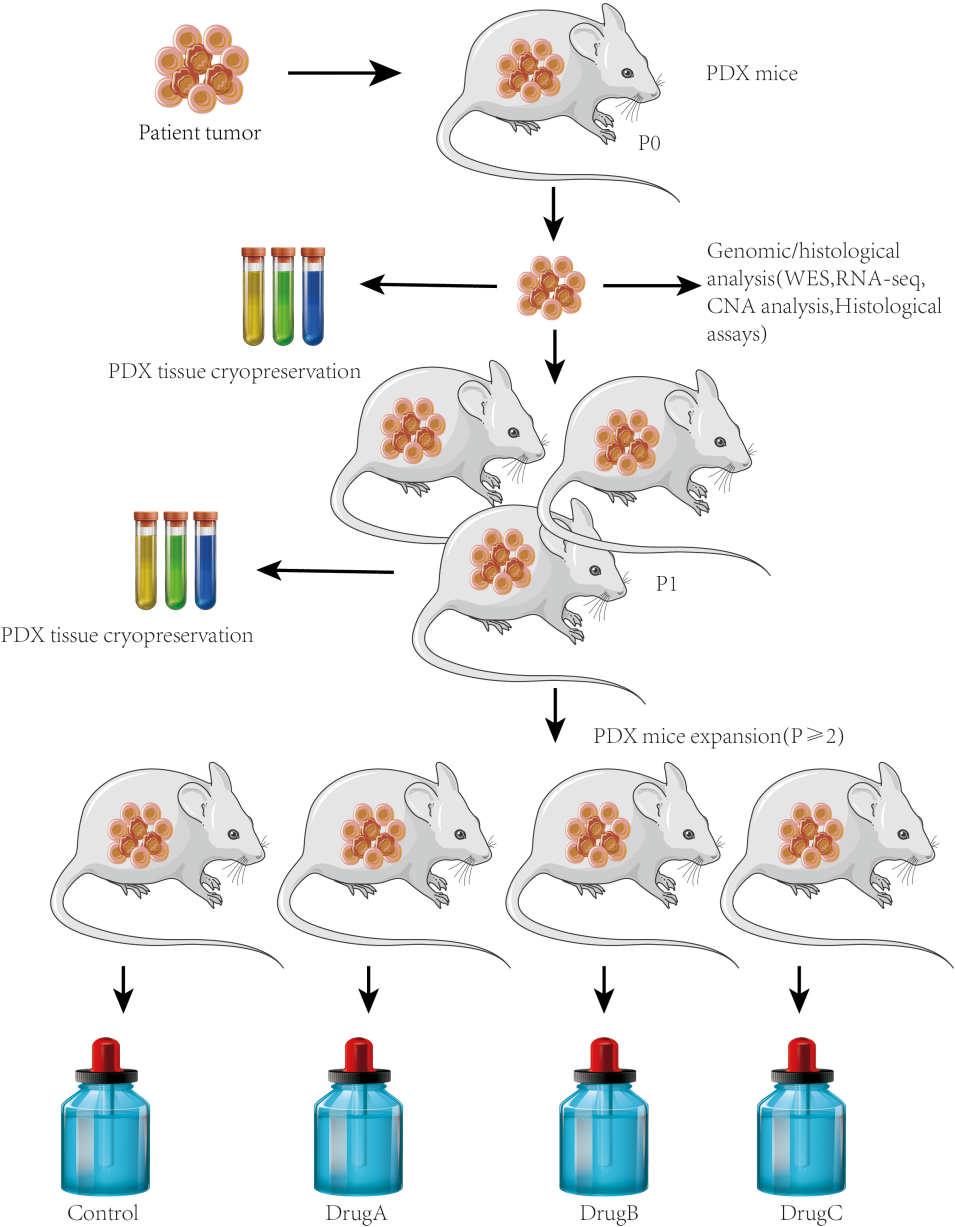
PDX models. Establishment of PDX models for metastasis research. Workflow showing the generation of PDX models from surgically resected human tumors. Tumor fragments were implanted subcutaneously or orthotopically into immunodeficient mice (e.g., NSG or NOD/SCID). Engrafted tumors were passaged into recipient mice and monitored for metastatic spread to distant organs. This model preserves patient tumor heterogeneity and enables the study of clinically relevant metastatic behavior.

PDX models begin with the collection of tumor tissue from cancer patients, typically obtained through biopsies or surgical resections. The patient-derived tumor tissue from primary tumors or metastatic sites is then implanted into immunodeficient mice including severe combined immunodeficiency (SCID), NOD/SCID, or NSG (NOD/SCID/IL2Rγnull) mice, which lack a functional immune system (**Figure 3**). The implanted tumor tissue can be subcutaneously implanted or orthotopically transplanted into the appropriate anatomical site to mimic the original tumor as a PDX model. The tumor survives, grows, and can be passed into subsequent generations of mice. Extensive characterization of PDX models using various molecular and histopathological techniques is required to confirm their fidelity to the original patient tumor (29).

Researchers can study the metastatic behavior of cancer cells. This includes monitoring the dissemination of cancer cells from the primary tumor site to distant organs or tissues, investigating the mechanisms of metastasis, and assessing the response to anti-metastatic therapies once the PDX models are established. PDX-based cancer metastasis models have been applied to evaluate the efficacy of potential therapeutic agents in a more clinically relevant setting. Treating the PDX models with different drugs or drug combinations helps researchers assess their impact on primary tumor growth and metastasis (30). Using PDX models to explore personalized medicine approaches, treatment responses in individual patient-derived models can guide treatment decisions (31).

PDX-based cancer metastasis models offer several advantages over traditional cell line-based models by maintaining the genetic and phenotypic heterogeneity of the original tumor, including the metastatic potential, making them more representative of patient tumors (32). These models provide a novel platform for researchers to study the complex interactions between cancer cells and the tumor microenvironment, and they have the potential to guide clinical decision-making and the development of targeted therapies for metastatic disease (33).

## Patient-derived organoid-based cancer metastasis models

Patient-derived organoid models or tumor organoids are three-dimensional cultures derived from patient tumor tissues that mimic the complexity and heterogeneity of the original tumor which have gained increasing attention in cancer research and are useful for studying various aspects of cancer, including metastasis. Tumor cells isolated from biopsies or surgical resections of patients are first embedded in a suitable matrix, such as Matrigel, and cultured in a specialized medium that supports their growth as three-dimensional organoids. This culture allows the cells to maintain their genetic and phenotypic characteristics, including their metastatic potential (34).

Manipulation of Patient-derived organoids is valuable in studying various steps of the metastatic cascade, including invasion, intravasation, circulation, extravasation, and colonization. Behavior analysis of cancer cells within the organoids enables researchers to gain insights into the molecular mechanisms underlying metastasis and identify potential therapeutic targets (35) and provides a valuable tool for studying cancer metastasis by offering a more physiologically relevant and patient-specific context compared to traditional cell line models (36).

## PDX-derived tumor cell line-based cancer metastasis models

PDX-derived tumor cell line models are established from patient-derived xenografts to study the metastatic behavior of cancer cells (37). PDX tumor cells can be isolated and cultured *in vitro* to establish cell lines (38) which are derived from the patient’s tumor and are representative of the tumor’s genetic and phenotypic features.

Researchers have applied PDX-derived tumor cells in various experimental setups (39), including *in vitro* migration and invasion assays and *in vivo* metastasis models. These systems allow monitoring of metastatic potential and evaluation of therapeutic responses (40).

**Figure 4:**
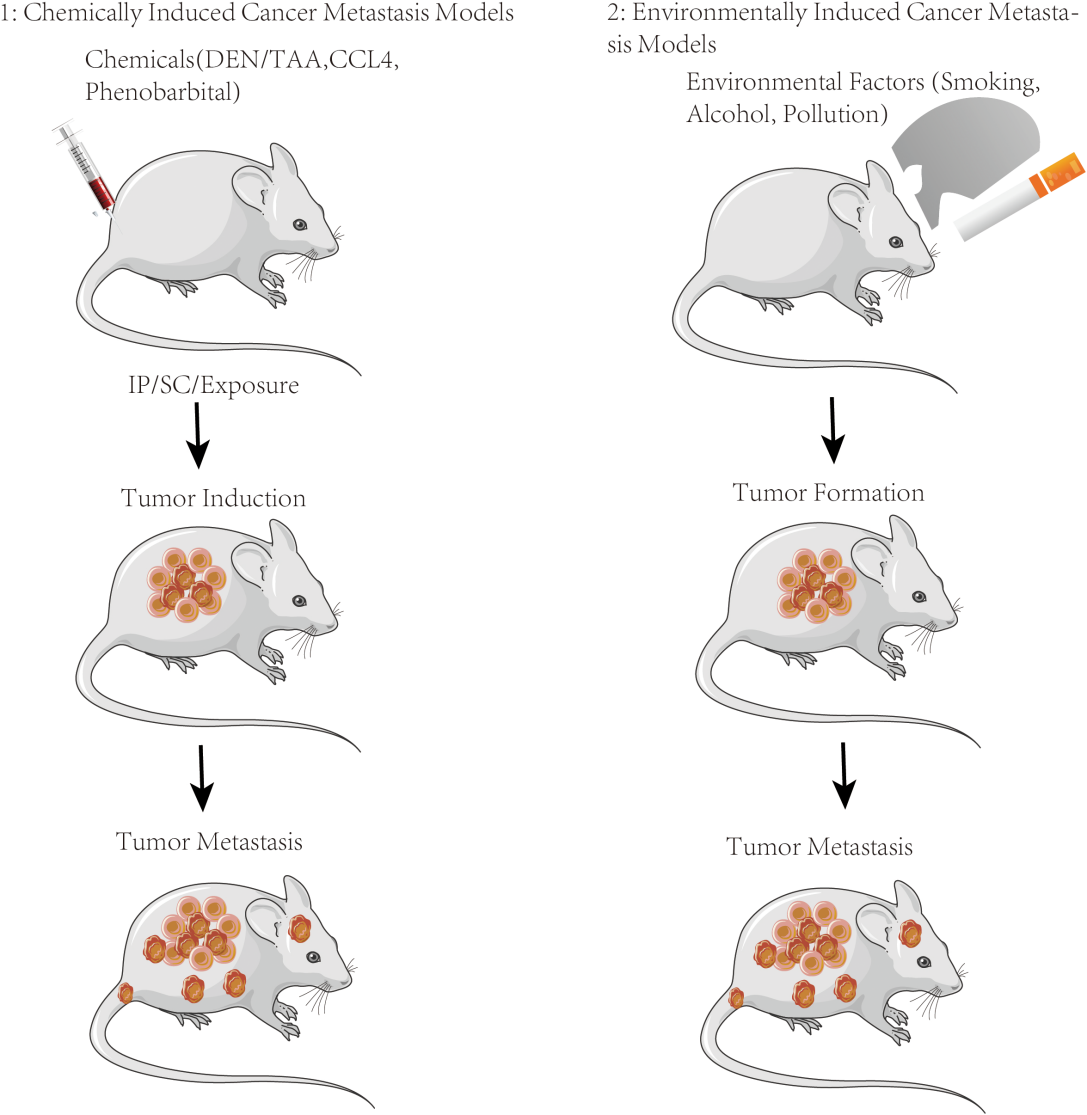
Metastasis induction animal models. Schematic of the chemical or environmental exposure-induced metastasis models used to evaluate the metastatic role *in vivo*.

PDX-derived tumor cell line-based models have both advantages of PDX models (retention of patient tumor characteristics) and *in vitro* cell line models (ease of manipulation and scalability) (41). Those models offer a valuable tool for studying the metastatic behavior of cancer cells and assessing potential therapeutic interventions in a controlled experimental setting. While PDX-derived tumor cell lines can provide important insights, they still belong to an *in vitro* model and may not fully recapitulate the complexity of the tumor microenvironment and the entire metastatic process seen *in vivo* (42) (**Figure 4**).

## Spontaneous genetically engineered mouse cancer metastasis models

GEM cancer metastasis models are specifically designed to study cancer metastasis which involves the manipulation of specific genes or signaling pathways in mice to recapitulate key aspects of human cancer metastasis (43) for understanding the molecular mechanisms underlying metastasis and testing novel therapeutic approaches (44).

Manipulation of specific genes or signaling pathways to mimic key features of human cancer metastasis induces the formation of primary tumors and subsequent metastasis to some organs or tissues in GEM models mimicking what occurs in human cancers (45–47). Tissue or organ-specific metastasis GEM models have been established in breast cancer metastasis research by targeting specific genes in the mammary gland to induce the development of primary breast tumors that subsequently metastasize to other organs, such as lungs or bones.

Metastasis models modified from GME provide an opportunity to investigate the molecular mechanisms underlying metastasis by studying the genetic alterations introduced in the mice and analyzing the resulting tumors and metastases in which researchers can identify critical genes and signaling pathways involved in different steps of the metastatic cascade (43). GEM cancer metastasis models are valuable for testing the efficacy of novel therapeutic interventions specifically targeting metastasis which involve administering targeted therapies, immunotherapies, or combination treatments to the mice and assessing their impact on primary tumor growth, metastasis, and overall survival (47). However, GEM models have limitations and may not fully recapitulate the entire complexity of human metastasis, a combination of GEM models with other experimental systems is often used to gain a comprehensive understanding of metastatic processes.

## Cancer cell line xenograft in cancer metastasis models

Cancer cell line xenograft models are commonly used in cancer metastasis studies which involve the transplantation of established cancer cell lines into immunocompromised mice or other animal hosts to investigate various aspects of metastasis (48). Cancer cell lines derived from human or animal tumors are selected based on their known metastatic potential or specific molecular characteristics associated with metastasis (39) and cultured *in vitro* and prepared for transplantation into animal hosts (40). Cancer cells are injected or implanted into immunocompromised mice, typically subcutaneously or orthotopically, to mimic the primary tumor site in a controlled *in vivo* environment (49).

Development of the primary xenograft tumors is crucial in assessing the dissemination of cancer cells from the primary site to distant organs or tissues, the colonization of secondary tumors, and the evaluation of metastatic burden. Xenograft tumors can be analyzed using molecular techniques to study the genetic and phenotypic features associated with metastasis which include genetic profiling, gene expression analysis, and examination of specific markers or signaling pathways involved in metastasis (50).

Application of cancer cell line xenograft models to evaluate the efficacy of therapeutic interventions targeting metastasis includes testing the impact of different drugs, treatment combinations, or experimental therapies on primary tumor growth and metastatic spread (51). Cancer cell line xenograft models are a simplified and controlled system for studying certain aspects of cancer metastasis allowing researchers to investigate the behavior of cancer cells *in vivo*, assess their metastatic potential, and evaluate therapeutic interventions (52). The limitations of these models, including potential differences from human tumors, lack of immune system interactions, and limited representation of the complex tumor microenvironment (48) make it essential to integrate cancer cell line xenograft models with other model systems to gain more comprehensive insights into cancer metastasis.

### Orthotopic cancer metastasis models

Orthotopic cancer metastasis models are developed from orthotopic models that involve the implantation of cancer cells into the organ or tissue of origin. One of those examples is that breast cancer cells can be injected into the mammary fat pad of mice to mimic breast cancer metastasis (53). These models can recapitulate the microenvironment and architecture of the primary tumor site, facilitating the study of organ-specific metastasis (54).

### Metastatic colonization models

To recreate and study specific steps of the metastatic process in a controlled laboratory setting, metastatic colonization models can help researchers investigate the mechanisms underlying tumor cell dissemination, colonization, and growth at distant sites which provide valuable insights into the molecular and cellular events that promote or inhibit metastasis, facilitating the development of new therapeutic strategies to target metastatic disease (55). In orthotopic models commonly used for metastatic colonization, tumor cells are injected directly into the organ or tissue from which the cancer originated. For instance, the injection of breast cancer cells into the mammary fat pad in mice allows researchers to study the ability of tumor cells to invade surrounding tissues, intravasate into the blood or lymphatic vessels, and establish metastatic colonies in the relevant organ (39). A more frequently used group of experimental metastasis models in which tumor cells are injected directly into the bloodstream or other systemic compartments, bypassing the initial steps of invasion and intravasation allows researchers to focus on the later stages of metastasis, such as the ability of tumor cells to survive in circulation, extravasate at distant sites, and form secondary tumors.

## Ex vivo cancer metastasis models

Ex vivo cancer metastasis models apply cultured tissues or organoids derived from patients or animal models to study the metastatic behavior of cancer cells which provide a controlled and manipulable environment to investigate various aspects of metastasis, such as the invasion of cancer cells, interactions with the surrounding microenvironment, and response to therapeutic agents (56). Ex vivo cancer metastasis models currently include organotypic slice cultures, organoid cultures, microfluidic platforms, and explant cultures (57).

Organotypic slice cultures are culturing thin slices of intact tumor or normal tissue ex vivo to maintain the architecture and cellular heterogeneity of the original tissue, allowing researchers to study the invasive behavior of cancer cells within a more physiologically relevant environment. The slices can be exposed to different factors or drugs to investigate their effects on metastasis (58).

Organoid cultures can initiate and sustain three-dimensional structures derived from stem cells or tissue samples capable of self-organizing and recapitulating the characteristics of the original tissue. Cancer organoids can be generated from patient-derived tumor samples or genetically engineered models providing a platform to study various aspects of metastasis, including invasion, intravasation, and colonization while retaining the genetic and phenotypic features of the primary tumor (59).

Microfluidic platforms as microfluidic devices offer a controlled and dynamic environment to mimic specific aspects of the metastatic process. These platforms are applied to recreate features of the tumor microenvironment, such as blood vessels, lymphatics, and extracellular matrix components so cancer cells can be introduced into these devices to study their migratory behavior, response to chemotactic signals, and interactions with different cell types (60).

Explant cultures can maintain small tissue fragments from tumors or metastatic sites ex vivo and be cultivated in various culture systems, including three-dimensional matrices or bioreactors, to investigate metastatic behavior. Explant cultures preserve the complex interactions between cancer cells and the surrounding stroma (61) while allowing researchers to monitor the growth and spread of cancer cells.

As a valuable tool for studying the biological processes of metastasis and evaluating potential therapeutic interventions, ex vivo cancer metastasis models offer a compromise between *in vivo* models being complex and less controlled, and *in vitro* cell culture models lacking the physiological context of the tumor microenvironment (62). Integrating ex vivo models with other experimental systems provides more comprehensive insights into the metastatic process and develops strategies to prevent or target metastasis more effectively.

## Cancer metastasis models with tissue or organ-specific

Tissue or organ-specific cancer metastasis models are essential for studying the unique aspects of metastasis in different tissues or organs, and they can mimic the microenvironment of a particular tissue or organ where metastasis occurs and incorporate key cellular and extracellular components, such as stromal cells, immune cells, and extracellular matrix components, that are specific to the tissue of interest (63). These models provide insights into the interactions between metastatic cancer cells and the surrounding tissue, which influence metastatic progression by recapitulating the tissue-specific microenvironment (64).

To unravel the underlying mechanisms of metastasis in different tissues or organs, tissue-specific metastasis models can be tailored to represent specific aspects of metastatic processes observed in tissues. For example, liver metastasis models may focus on understanding the interactions between cancer cells and hepatocytes, meanwhile, models of lung metastasis may refer to the mechanisms of extravasation and interaction with lung epithelial cells. Tissue or organ-specific metastasis models are valuable in evaluating the efficacy of therapeutic strategies targeting tissue-specific metastases if a particular tissue is known to have unique signaling pathways or immune responses (65).

Tissue-specific metastasis models which improve the characterization and evaluation of biomarkers specific to tissues or organs can be adapted to assess the expression or functions of tissue-specific biomarkers in metastatic tumors. Metastasis models with tissue or organ specificity enable the further analysis of tissue-specific immune responses and immunomodulatory factors to elucidate the interactions between metastatic cancer cells and immune cells within the tissue microenvironment for identifying potential targets for immunotherapies and develop strategies to enhance anti-tumor immune responses in specific tissues (66).

Tissue or organ-specific metastasis models facilitate the translation of preclinical findings to clinical practice. They provide platforms for studying tissue-specific metastasis and evaluating the efficacy of tissue-specific treatment approaches. The knowledge gained from these models can inform clinical decision-making, treatment selection, and the design of clinical trials for patients with tissue-specific metastases (66).

Cancer metastasis models with tissue or organ-specific characteristics are critical in understanding the unique aspects of metastasis in different tissues or organs which help recapitulate tissue-specific microenvironments, investigate tissue-specific metastatic mechanisms, evaluate tissue-specific therapeutic strategies, assess tissue-specific biomarkers, examine tissue-specific immune responses, and facilitate the translational impact of research on tissue-specific metastasis (67).Among various organ-specific metastases, bone metastasis represents a particularly well-characterized and clinically relevant model.

## Mechanisms of bone metastasis

Bone metastasis is a complex, multistep process involving the colonization and growth of cancer cells within the bone microenvironment, dramatically contributing to disease progression and poor clinical outcomes. This process is characterized by a dynamic and reciprocal interaction between metastatic cancer cells and the bone microenvironment, commonly referred to as the “vicious cycle” of bone metastasis (68). Following dissemination from the primary tumor, cancer cells that reach the bone must first survive within the unique bone microenvironment. They adhere to the bone matrix and interact with resident cells, including osteoblasts, osteoclasts, bone marrow stromal cells, and immune cells (69). Once established, metastatic cells disrupt the normal balance between bone formation and resorption.

In osteolytic bone metastases, which are typical of breast cancer and some lung cancers, tumor cells secrete factors such as parathyroid hormone-related protein (PTHrP), interleukins (e.g., IL-6, IL-11), and other osteoclast-activating factors (70). These stimulate osteoblasts to express receptor activator of nuclear factor kappa-β ligand (RANKL), which in turn promotes osteoclast differentiation and activation. The resulting bone resorption releases growth factors stored in the bone matrix, such as transforming growth factor-beta (TGF-β) and insulin-like growth factors (IGFs), which further stimulate tumor growth, thereby perpetuating the vicious cycle. Conversely, osteoblastic bone metastases, commonly seen in prostate cancer, are associated with excessive bone formation (71). Tumor-derived factors such as endothelin-1 and bone morphogenetic proteins (BMPs) stimulate osteoblast activity, leading to abnormal bone deposition. However, this newly formed bone is often structurally weak and disorganized, contributing to skeletal complications.

## Bone-specific metastasis models

**Figure 5:**
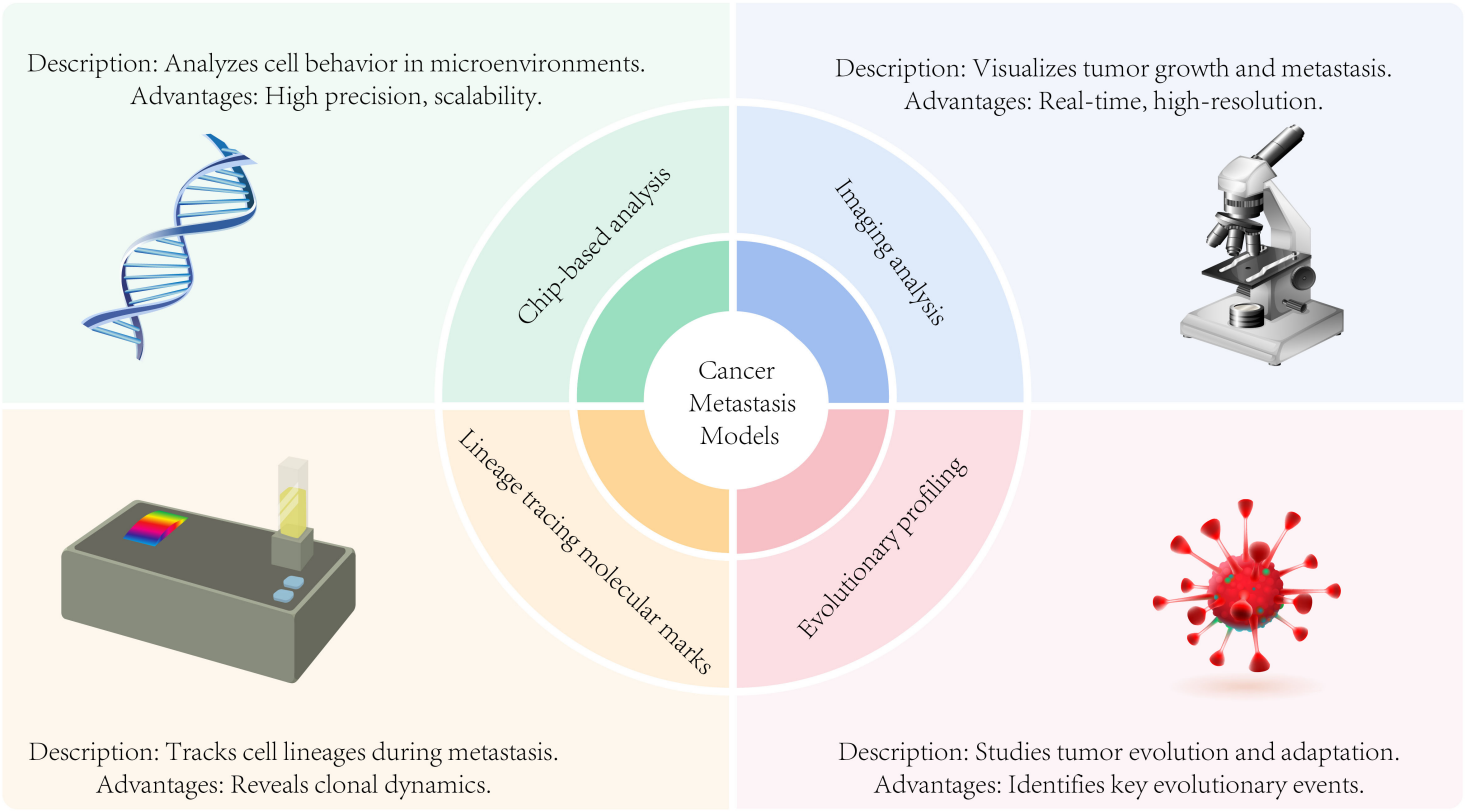
Analysis modes of metastasis models including chip-based analysis, imaging analysis, evolutionary profiling, and lineage tracing molecular marks.

Experimental models are essential tools for dissecting the complex interactions between cancer cells and the bone microenvironment, as well as for evaluating potential therapeutic strategies. *In vitro* models, including 2D co-culture systems, 3D spheroids, and organ-on-a-chip platforms, allow controlled studies of cancer–bone cell interactions and early metastatic steps (72). *In vivo* models, such as intratibial injection, intracardiac injection, or orthotopic models in mice, recapitulate various stages of bone colonization and the development of osteolytic or osteoblastic lesions, providing insights into disease progression and therapeutic responses. Importantly, the integration of complementary *in vitro* and *in vivo* models is crucial to overcome the limitations of individual systems and to generate a more comprehensive understanding of bone metastasis. Such integrative approaches can more accurately mimic the complex biology of the bone metastatic niche, helping to identify molecular drivers of metastasis and to develop clinically relevant therapeutic strategies (**Figure 5**).

## Analysis modes of metastasis models

### Chip-based analysis of cancer metastasis models

Chip-based analysis utilizes microfluidic or lab-on-a-chip devices to study cancer metastasis within a miniaturized and precisely controlled environment, enabling efficient investigation of key metastatic processes (73). These platforms offer researchers precise control over biological samples and experimental conditions, allowing for systematic exploration of various aspects of cancer metastasis.

Microfluidic devices can be engineered to replicate essential features of the tumor microenvironment, including vasculature, lymphatics, and extracellular matrix components (74). This allows researchers to study cancer cell interactions with surrounding immune cells, stromal cells, and signaling molecules in a physiologically relevant and highly controllable setting.

Cell migration and invasion, and microfluidic devices are the normal available options to assess the migration and invasion of cancer cells. The ability of cancer cells to migrate through confined spaces, cross endothelial barriers, or invade three-dimensional matrices can be investigated through the precise control of flow conditions in understanding the mechanisms involved in cancer cell dissemination during metastasis.

Analyzing circulating tumor cells (CTCs), microfluidic devices can capture and analyze circulating tumor cells (CTCs) present in the bloodstream of cancer patients or animal models. These devices can isolate CTCs from blood samples, allowing their characterization and study to provide insights into the metastatic potential of cancer cells and help monitor disease progression (75).

Microfluidic devices can be utilized for high-throughput drug screening to evaluate the efficacy of potential therapeutic agents on cancer metastasis for drug screening and therapeutic testing (76). The response of metastatic cells to different drugs or drug combinations can be examined by incorporating cancer cells and relevant components of the tumor microenvironment into the chip in the identification of targeted therapies or personalized treatment strategies.

Microfluidic devices are emerging as powerful tools for isolating and analyzing single cells from metastatic tumors (77). These platforms enable the precise capture of individual cancer cells, facilitating detailed molecular profiling, including gene expression analysis, DNA sequencing, and proteomic studies. By replicating essential features of the tumor microenvironment—such as vasculature, lymphatics, and extracellular matrix—microfluidic devices also allow researchers to investigate cancer cell interactions with immune cells, stromal cells, and signaling molecules in a physiologically relevant setting.

Single-cell analysis represents a state-of-the-art approach for uncovering cellular heterogeneity within metastatic tumors. It enables the identification of specific subpopulations and molecular signatures associated with metastatic potential and disease progression (78).

The advantages of chip-based analysis in cancer metastasis research lie in its precise control, scalability, and ability to mimic key aspects of the tumor microenvironment. Although these technologies are still rapidly evolving, they offer significant potential for studying diverse aspects of metastasis, including cellular behavior, microenvironmental interactions, and responses to therapeutic interventions. Ultimately, chip-based platforms contribute to a deeper understanding of metastatic mechanisms and hold promise for guiding the development of more effective, targeted therapeutic strategies.

### Imaging analysis in cancer metastasis models

Imaging analysis provides valuable insights into the behavior and progression of metastatic lesions in the study of cancer metastasis models. Various imaging techniques are also developed to visualize, characterize, and monitor metastatic lesions in preclinical models. So far Imaging modalities like bioluminescence imaging (BLI), fluorescence imaging, positron emission tomography (PET) imaging, magnetic resonance imaging (MRI), computed tomography (CT) imaging, and intravital microscopy have been commonly applied in cancer metastasis research (79).

BLI uses luciferase-expressing cancer cells or reporter genes to track tumor growth and metastasis in live animals in which luciferase activity is visualized by injecting a substrate, which emits light that can be captured and quantified using specialized cameras. BLI offers longitudinal monitoring of metastatic lesions and enables researchers to assess the response to therapeutic interventions (80).

Fluorescence imaging techniques including fluorescence microscopy and fluorescent molecular imaging utilize fluorescent probes or dyes to visualize specific molecules or structures of interest. Metastatic lesions and the assessment of their distribution and size can be visualized by fluorescently labeled antibodies or molecular probes targeting metastatic markers (81).

PET imaging involves the injection of radiolabeled tracers that are taken up by metastatic lesions in which the emitted positrons are detected by specialized scanners, generating three-dimensional images of the tumor burden. The combination of PET imaging with CT or MRI greatly improves the collection of anatomical context and more detailed information about the metastatic lesions (82).

By utilizing powerful magnets and radiofrequency pulses to generate detailed images of the body’s internal structures, MRI can provide high-resolution anatomical images of metastatic lesions, aiding in their detection and characterization. Functional MRI techniques like diffusion-weighted and dynamic contrast-enhanced MRI can provide information about tissue microstructure and perfusion for studying metastasis.

CT imaging uses X-rays to generate cross-sectional images of the body to provide detailed anatomical information in analysis of the size, location, and progression of metastatic lesions. CT imaging combined with contrast agents enhances the visualization of blood vessels and tumor perfusion (83).

Intravital microscopy as one of the specialized microscopy techniques to visualize metastatic processes in live animals enables real-time imaging of cellular behavior, interactions, and dynamic processes within the tumor microenvironment (84). Intravital microscopy can take valuable insights into the mechanisms of metastasis, including tumor cell migration, invasion, and interactions with the surrounding stroma.

All these imaging modalities contribute to our understanding of cancer metastasis by allowing non-invasive visualization and characterization of metastatic lesions in preclinical models which aid in assessing tumor burden, monitoring disease progression, evaluating therapeutic responses, and uncovering the underlying mechanisms of metastasis. The smart combination of all those advanced image analysis techniques will produce more quantitative and qualitative information critical for studying cancer metastasis.

## Lineage tracing molecular marks in cancer metastasis models

Lineage tracing molecular marks utilized in cancer metastasis models to track and identify the progeny of specific cells or lineages during the metastatic process enables researchers to understand the clonal dynamics, cellular heterogeneity, and fate of individual cells during metastasis (85). Lineage tracing in cancer metastasis models includes genetic lineage tracing, fluorescent protein labeling, barcoding, Single-cell RNA sequencing, and various lineage-specific reporter systems.

Genetic lineage tracing introduces genetic markers or reporters into specific cell populations by using genetically engineered mice or viral vectors to express fluorescent proteins, such as GFP (green fluorescent protein), or enzymes capable of permanently labeling cells, such as Cre recombinase or Flp recombinase (86). Crossing these mice with cancer models or inducing the expression of the markers in specific cells helps track the descendants of marked cells during metastasis.

Fluorescent proteins, such as GFP, can be expressed under the control of specific promoters or genetic elements associated with metastatic cells. By introducing these markers into cancer cells or using them to label specific populations of cells, researchers can track the migration, invasion, and colonization of these cells during metastasis (87).

Barcoding is to introduce unique DNA or RNA sequences into individual cell clones labeled with a specific barcode, allowing the identification, and tracking of their progeny during metastasis through applying viral vectors or CRISPR-based techniques to introduce the barcodes into cancer cells. Barcoding with high-throughput sequencing technologies is the most used method for analysis of the metastatic potential and clonal evolution of individual cancer cells (88).

Single-cell RNA sequencing (scRNA-seq) profiles gene expression at the single-cell level. Sequencing the RNA from individual cells helps identify the transcriptional signatures of specific cell lineages or clones during metastasis and provides insights into the cellular heterogeneity and differentiation trajectories of metastatic cells (89).

Various lineage-specific reporter systems are employed to trace the progeny of specific cell types during metastasis by using genetic elements or promoters that are active only in certain cell lineages or stages of development. The metastatic potential of cell populations can be selectively labeled and tracked by driving the expression of reporters or markers under these lineage-specific promoters, researchers. Lineage tracing molecular marks helps trace the origin, dynamics, and fate of cancer cells during metastasis and uncover the clonal evolution, cellular heterogeneity, and mechanisms underlying metastatic progression (90). Lineage tracing with advanced molecular and imaging approaches allows for a deeper understanding of the metastatic process and may contribute to the development of targeted therapies against metastatic disease (91).

## Evolutionarily profiling the cancer metastasis models

Evolutionary profiling of cancer metastasis models involves studying the dynamic changes and evolutionary processes that occur during metastasis. This profiling aims to understand how tumors evolve, acquire metastatic properties, and adapt to new microenvironments. Some approaches for evolutionarily profiling cancer metastasis models include genetic profiling, studying clonal dynamics and heterogeneity, tumor microenvironment characterization, functional profiling, imaging-based profiling, and integrating multi-omics data (92).

Genetic profiling of genomic alterations in primary tumors and metastatic lesions, such as mutations, copy number variations, and chromosomal rearrangements, is crucial in identifying genetic changes contributing to metastasis and assessing the clonal evolution of metastatic cells.

Analysis of clonal dynamics and heterogeneity of the subpopulations of cells contribute to metastasis. Techniques like single-cell sequencing, bulk sequencing, and lineage tracing are available to detect the clonal composition, genetic diversity, and evolutionary trajectories in metastatic tumors to understand the cellular dynamics and selection pressures during metastasis.

Evolutionary profiling of the tumor microenvironment in metastasis involves characterizing the interactions between cancer cells and the surrounding stromal cells, immune cells, and extracellular matrix components which includes assessing the presence and activation state of immune cells, analyzing the composition of the extracellular matrix, and evaluating angiogenesis and hypoxia within metastatic lesions. Functional profiling of functional changes associated with metastasis includes analyzing alterations in signaling pathways, gene expression profiles, epigenetic modifications, and metabolic changes. Profiling of functional genomics, proteomics, and metabolomics, provides insights into the molecular mechanisms underlying metastasis (93).

MRI, PET, optical imaging, and other imaging techniques utilized to profile the spatial and temporal dynamics of metastatic lesions allow researchers to monitor tumor growth, visualize the distribution of metastatic lesions, and assess the response to therapeutic interventions. Advanced imaging approaches like multiplexed imaging and intravital microscopy can confer more high-resolution characterization of the tumor microenvironment and cellular interactions (94).

Integrating multi-omics data including genomic, transcriptomic, proteomic, and epigenomic data can acquire a comprehensive view of the evolutionary landscape of metastatic tumors in identifying key driver events, pathways, and regulatory networks associated with metastasis (95). Evolutionarily profiling cancer metastasis models provides a deeper understanding of the molecular and cellular changes that occur during metastasis. All this knowledge can guide the development of targeted therapies, identify biomarkers of metastatic progression, and offer insights into strategies for prevention and treatment of metastatic disease (96).

## Metastasis induction models

### Chemically induced cancer metastasis models

Chemically induced cancer metastasis models involving the administration of specific chemical agents or treatments to induce metastatic lesions in animal models are used to study the mechanisms of metastasis and evaluate potential therapeutic interventions. Carcinogens from chemical substances induce the development of tumors and metastatic tumors in animal models which involve exposing animals to known carcinogens, such as tobacco smoke, chemical mutagens, or specific compounds, to initiate the formation of primary tumors that can subsequently metastasize. The carcinogen-induced models employed in various animal species include mice, rats, and other relevant models.

Certain chemicals or compounds can promote tumor growth and facilitate metastasis. For example, phorbol esters, such as 12-O-tetradecanoylphorbol-13-acetate (TPA), are known to induce inflammation and enhance metastatic potential in experimental models. These tumor-promoting agents are applied topically or systemically to initiate or facilitate the metastatic process in animal models.

Angiogenesis, forming new blood vessels, plays a crucial role in tumor growth and metastasis. Some chemical compounds, such as vascular endothelial growth factor (VEGF) or fibroblast growth factor (FGF) analogs, can induce angiogenesis and promote the growth of metastatic lesions. These compounds are administered to animal models to mimic the angiogenic process seen in cancer metastasis (97).

The immune system plays a critical role in regulating cancer metastasis. Certain chemical agents, such as immunosuppressive drugs or immune-modulating compounds, can manipulate the immune response and facilitate metastasis in animal models. These models help researchers investigate the interplay between the immune system and metastatic cells (98).

Metastasis-promoting treatments apply chemotherapeutic agents or radiation treatments primarily targeting primary tumors to influence the metastatic process. These treatments may induce DNA damage or alter the tumor microenvironment, promoting the development of metastatic lesions. Animal models are the best choice utilized to study the impact of these treatments on metastasis and explore potential strategies to mitigate metastatic spread (99). Chemically induced cancer metastasis models provide controlled and reproducible systems for studying the metastatic process and evaluating the efficacy of therapeutic interventions. However, the values of these models are restricted by the specific mechanisms targeted by the chemicals used and the potential differences from naturally occurring metastasis in human patients. Therefore, chemically induced models combined with other metastasis models such as genetically engineered models or patient-derived models will elicit a more comprehensive understanding of cancer metastasis (100).

### Environmentally induced cancer metastasis models

Environmentally induced cancer metastasis models that expose animals to specific environmental factors or conditions to induce tumor metastasis mimic environmental exposure conditions that are associated with an increased risk of cancer metastasis in humans. Chronic inflammation is associated with an increased risk of cancer development and metastasis. Animal models can be achieved through the administration of pro-inflammatory agents, such as lipopolysaccharides (LPS), or by genetic manipulation to generate chronic inflammatory conditions (101).

Diet and nutrition also contribute to cancer development and progression, including metastasis. The specific dietary factors such as high-fat diets, high-sugar diets, or diets deficient in certain nutrients affect tumor growth and metastasis in animal models which provide insights into the relationship between diet, metabolism, and metastatic processes (102).

It is known that exposure to environmental pollutants and toxins has been associated with increased cancer risk, including metastasis. Animal models exposing specific pollutants such as cigarette smoke, industrial chemicals, or air pollutants allow researchers to investigate the mechanisms by which environmental factors contribute to the metastatic process (103).

Hypoxia, or low oxygen levels as a common feature of the tumor microenvironment is associated with increased metastatic potential. Animal models by manipulating oxygen levels in the environment or by introducing hypoxia-inducible factors enable researchers to study the impact of hypoxia on tumor growth, angiogenesis, and metastasis (104).

Physical factors such as mechanical forces, radiation, or temperature changes can alter tumor progression and metastasis. Animal models subjected to specific physical conditions such as mechanical compression, ionizing radiation, or temperature variations, are applied to assess their effects on tumor behavior and metastasis which help in understanding the role of physical forces in the metastatic process (105).

Environmentally induced cancer metastasis models are controlled approaches to study the impact of specific environmental factors on metastasis. However, these models are limited to impact factors such as the complexity of human exposures and potential differences between animal models and human responses. Therefore, environmentally induced models combined with other metastasis models including genetically engineered models or patient-derived models can promote a more comprehensive understanding of cancer metastasis in the context of environmental influences (106).

### Clinical lab-assisted metastasis prediction, monitoring, and treatment

Clinical laboratory-assisted metastasis prediction and monitoring involving various laboratory tests and biomarkers are important to assess the risk of metastasis, detect metastatic lesions, and monitor the development of metastatic disease in cancer patients while providing valuable information for prognosis, treatment decision-making, and evaluating therapeutic response. Imaging techniques like CT, MRI, PET, or bone scans for detecting the presence and extent of metastatic lesions in different organs or tissues (107) help identify metastatic sites and monitor changes in tumor burden over time.

Tumor markers produced by cancer cells or normal cells in response to cancer such as prostate-specific antigen (PSA) for prostate cancer or carcinoembryonic antigen (CEA) for colorectal cancer can indicate the presence of metastasis or monitor treatment response (108). Blood tests are the most efficient way to measure the levels of these tumor markers and track their changes over time (109).

Circulating tumor cells (CTCs) are cancer cells detached from the primary tumor entering the bloodstream. Detection and enumeration of CTCs in peripheral blood samples can acquire substantial information about the presence of disseminated cancer cells and the risk of metastasis by technologies such as CTC isolation platforms, immunostaining, or molecular analysis employed to capture and analyze CTCs (110).

Circulating tumor DNA (ctDNA) referring to the small fragments of DNA released by tumor cells into the bloodstream can be analyzed through liquid biopsies by detecting specific genetic alterations or mutations associated with metastatic disease. ctDNA analysis can depict the molecular characteristics of metastatic lesions and monitor treatment response or disease progression.

Biomarker profiling of various biomolecules such as proteins, nucleic acids, or metabolites helps identify specific markers associated with metastasis by applying techniques such as gene expression profiling, next-generation sequencing, proteomics, or metabolomics to identify and validate biomarkers that predict the likelihood of metastasis or monitor metastatic progression (111).

Histopathological examination of biopsy or surgical specimens remains an essential and practical tool for detecting metastatic lesions. Pathologists examine the tissue samples under a microscope to identify microscopic features indicative of metastasis (112). Immunohistochemistry can be used to assess specific protein expression patterns associated with metastatic potential.

Molecular imaging: Molecular imaging techniques, such as positron emission tomography (PET) combined with specific radiotracers or contrast agents, can provide functional and molecular information about metastatic lesions. Molecular imaging can identify specific molecular targets or biomarkers associated with metastasis and aid in treatment planning and monitoring (113).

**Figure 6:**
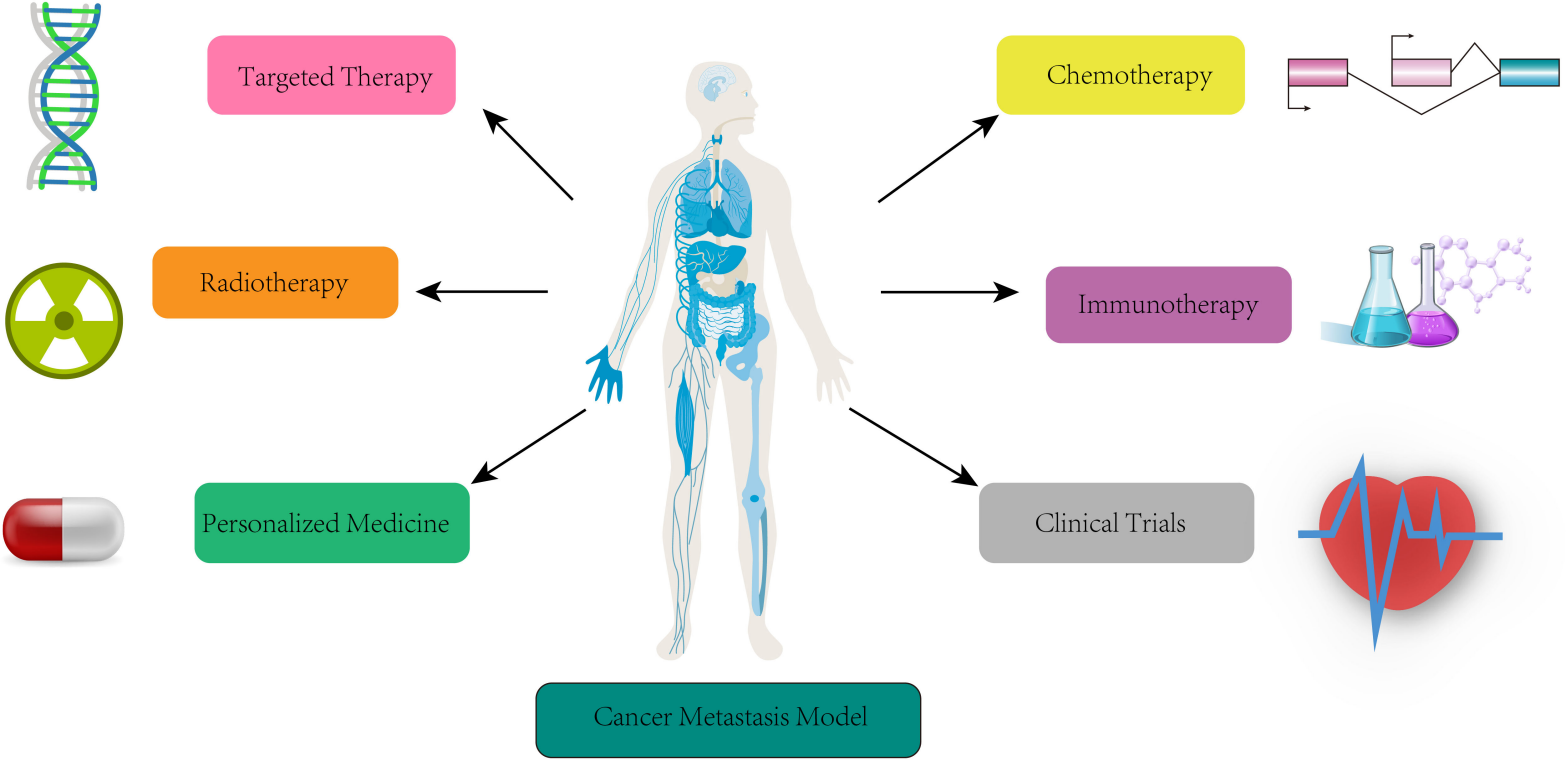
Clinical lab-assisted metastasis models for prediction, monitoring and treatment. Summary of clinical applications of metastasis models in target therapy, chemotherapy, radiotherapy, immunotherapy, personal medicine, and clinical trials.

Clinical laboratory-assisted metastasis prediction and monitoring provide valuable information for patient management and treatment decisions. These laboratory tests and biomarkers contribute to early detection, prognostication, and evaluation of therapeutic responses in metastatic cancer patients (114). Integrating multiple laboratory approaches can provide a comprehensive assessment of metastatic disease and guide personalized treatment strategies (**Figure 6**).

### Cancer metastasis models for targeted therapy

Cancer metastasis models are instrumental in the development and implementation of targeted therapies for metastatic cancer being crucial in dissecting the mechanisms of metastasis and aiding in the identification and optimization of targeted treatment strategies. Cancer metastasis models improve targeted therapy through understanding the molecular basis of metastasis, target identification and validation, preclinical evaluation of targeted therapies, optimization of treatment strategies, study of resistance mechanisms, personalized treatment selection, and facilitation of translational impact (115). Metastasis models help elucidate the molecular alterations and signaling pathways involved in metastatic progression by simulating the interactions between cancer cells, the tumor microenvironment, and the host immune system (116). By integrating patient-specific data such as genomic information and molecular profiling, metastasis research models can identify genetic mutations, altered signaling pathways, or overexpressed proteins critical for metastasis. Target validation in metastasis models helps prioritize, select, and evaluate the most promising candidates for further development as targeted therapies (117).

Cancer metastasis models allow researchers to simulate the response of metastatic tumors to targeted agents, including small molecule inhibitors, monoclonal antibodies, and immunotherapies by assessing factors such as tumor regression, inhibition of metastatic spread, and survival outcomes on the effectiveness of targeted therapies in a metastatic setting. Models for cancer metastasis improve the optimization of treatment strategies for targeted therapy and help evaluate the effectiveness of different treatment regimens, including dose optimization, treatment schedules, and combination therapies. These models simulate the response of metastatic tumors to various treatment scenarios to guide the selection of optimal treatment strategies that maximize therapeutic efficacy and minimize the development of resistance (118).

Translational metastasis models facilitate the studying the mechanisms of resistance to targeted therapies by simulating the evolution of cancer cells under selective pressure from targeted agents. These models can predict the emergence of resistant clones, identify potential resistance mechanisms, and help improve strategies to overcome or prevent resistance, such as combination therapies or alternative target identification.

Metastatic cancer models with patient-specific data contribute to personalized treatment selection for targeted therapy by integrating genomic and molecular information from individual patients and simulating the response of metastatic tumors to different targeted agents. Those metastasis models bridge the gap between preclinical research and clinical practice by providing a platform for translational studies and enabling clinicians to make informed decisions about the most effective targeted therapies for each patient based on their unique molecular profile and metastatic characteristics (119).

### Cancer metastasis models for chemotherapy

Cancer metastasis models are crucial in understanding the response of metastatic tumors to chemotherapeutic agents and optimizing treatment strategies by evaluating drug efficacy, optimizing treatment regimens, studying drug resistance mechanisms, enabling personalized treatment selection, predicting treatment response, optimizing combination therapies, and facilitating the translational impact of chemotherapy research. Application of these models enhances the effectiveness of chemotherapy treatments and improves outcomes for patients with metastatic cancer (120).

### Cancer metastasis models for radiotherapy

Cancer metastasis models are valuable tools for studying the effects of radiotherapy on metastatic tumors which strengthen understanding of the response of metastatic tumors to radiation therapy, contribute to treatment planning in radiotherapy, provide a platform for evaluating treatment response to radiotherapy, enable the evaluation of combination therapies involving radiotherapy, assist with studying the mechanisms of radiation resistance that arise during radiotherapy, and guide personalized treatment selection in radiotherapy (121). These models enhance the efficiency of radiotherapy and improve outcomes for patients with metastatic cancer.

### Cancer metastasis models for immunotherapy

Cancer metastasis models for immunotherapy specifically contribute to understanding immune responses, predicting treatment responses, optimizing combination therapies, studying resistance mechanisms, and identifying biomarkers (122). Metastasis models help analyze the complex interactions between the immune system and metastatic tumors. These models Simulate the immune response within the tumor microenvironment to interrogate the mechanisms of immune evasion and tumor immune surveillance and identify factors that influence the effectiveness of immunotherapy in metastatic cancer (123).

Molecular profiles of tumor metastasis models help predict the response of individual patients to immunotherapy. Incorporating patient-specific data such as immune cell profiles, tumor antigen expression, and immune checkpoint expression into the models can simulate the response of metastatic tumors to different immunotherapeutic agents which clinicians in selecting the most effective immunotherapies for each patient based on their unique molecular profile and metastatic characteristics.

Cancer metastasis models can simulate the effects of combining immunotherapeutic agents with other treatment modalities, such as chemotherapy, radiation therapy, or targeted therapies by assessing the response of metastatic tumors to various combination therapy scenarios to optimize treatment approaches and identify synergistic interactions between immunotherapy and other treatments (124). For studying the mechanisms of resistance to immunotherapy, these models can simulate the evolution of cancer cells under selective pressure from immunotherapeutic agents to predict the emergence of resistant clones and identify potential resistance mechanisms to overcome or prevent immunotherapy resistance, such as combination therapies or identification of alternative treatment approaches (125). Metastasis models integrate genomic and molecular information from individual patients to identify potential predictive biomarkers, such as tumor mutational burden, immune cell infiltration patterns, or expression levels of immune checkpoint molecules which help select patients more likely to benefit from immunotherapy.

Metastasis models with patient-specific data contribute to personalized treatment selection in immunotherapy by integrating genomic and molecular information from individual patients. These models can simulate the response of metastatic tumors to different immunotherapeutic agents and enable clinicians to make informed decisions about the most effective immunotherapies for each patient based on their unique molecular profile and metastatic characteristics (126).

Metastasis models can bridge the gap between preclinical research and clinical practice in immunotherapy, provide a platform for the preclinical evaluation of immunotherapeutic agents, and facilitate the translation of promising agents from the laboratory to clinical trials (127). Cancer metastasis models not only contribute to understanding immune responses, predicting treatment responses, optimizing combination therapies, studying resistance mechanisms, identifying biomarkers, enabling personalized treatment selection, and facilitating the translational impact of immunotherapy research but also enhance the effectiveness of immunotherapies and improve outcomes for patients with metastatic cancer (128).

### Cancer metastasis models for personalized medicine

For personalized medicine, cancer metastasis models enable a better understanding of the complex processes involved in metastasis and aid in developing personalized treatment strategies via predicting metastatic potential, identifying therapeutic targets, evaluating treatment efficacy, optimizing treatment combinations, assessing treatment resistance, translating preclinical findings, and personalizing clinical trial design. Incorporating patient-specific data such as tumor characteristics, genetic mutations, and molecular profiles helps analyze the metastatic potential of a tumor (100). Cancer metastasis models can identify key genes, signaling pathways, and cellular processes involved in metastasis by simulating the interactions between cancer cells, the tumor microenvironment (129), and the host immune system which helps in selecting personalized therapies that target these specific drivers of metastasis. Tumor metastasis models can also be used to evaluate the effectiveness of different treatment strategies and predict the treatment response in individual patients.

Metastatic tumors often exhibit complex molecular profiles and resistance mechanisms and can simulate the effects of different drug combinations to identify synergistic treatment approaches (130). These models can predict the emergence of resistant clones and identify potential resistance mechanisms by simulating the evolution of cancer cells and their response to treatments which aids in devising personalized treatment strategies to overcome or prevent treatment resistance.

Tumor metastasis models provide a platform to evaluate the efficacy and safety of potential therapeutic interventions before they are tested in human clinical trials and ensure that personalized treatment approaches are built upon robust evidence from preclinical models (131). Metastasis models assist in designing trials that target specific subtypes of metastatic tumors by simulating different patient populations, treatment protocols, and response criteria, these models (132). Playing a vital role in personalized medicine, cancer metastasis models are needed in predicting metastatic potential, identifying therapeutic targets, evaluating treatment efficacy, optimizing treatment combinations, assessing treatment resistance, translating preclinical findings, and informing personalized clinical trial design.

### Cancer metastasis models for clinical trial

As valuable tools in the context of clinical trials for studying the efficacy and safety of potential treatments for metastatic cancer, cancer metastasis models contribute to clinical trials by involving preclinical evaluation, treatment optimization, patient selection and stratification, biomarker identification, predicting treatment response, safety assessment, and translational impact. These models are currently applied to assess the efficacy and safety of novel therapies in a controlled and reproducible manner, identify promising treatments, and guide decision-making regarding their progression to clinical trials (133). These models also help select optimal treatment approaches by simulating the response of metastatic tumors to different treatment regimens including dosing, timing, and combination therapies.

Modeling cancer metastasis can simulate the response of metastatic tumors in different patient populations by integrating patient-specific data, such as molecular profiles, clinical characteristics, and metastatic sites (134). Model establishing for cancer metastasis can predict treatment response or detect disease progression in clinical trials via identifying and validating biomarkers. Tumor metastasis models can predict treatment response to specific therapies based on patient-specific characteristics by integrating patient-specific data into the models, such as genomic profiles, molecular alterations, and tumor microenvironment characteristics (135).

Clinical metastasis models can help identify potential toxicities and guide the determination of safe dosage levels by evaluating the effects of therapies on normal tissues, organs, and the immune system. Safety assessment in metastasis models helps reduce patient risks during clinical trial development (136). Clinical-relevant metastasis models bridge the gap between preclinical research and clinical practice by providing a platform for evaluating potential treatments in a metastatic context. The information gained from these models will improve the design and implementation of clinical trials and ensure that promising treatments identified in preclinical studies are appropriately translated to the clinical setting (137).

Cancer metastasis models can provide preclinical evaluation, optimize treatment strategies, aid in patient selection and stratification, identify biomarkers, predict treatment response, assess safety (138), and facilitate the translational impact of research which enhances the effectiveness and efficiency of clinical trials for metastatic cancer, ultimately improving patient outcomes.

## The future of cancer metastasis models

The future of cancer metastasis models will provide more exciting promise and potential for advancing our understanding of cancer progression and developing effective treatments. Since cancer metastasis models will continue to evolve and become more accurate and sophisticated, advances in new technologies like computational modeling, machine learning, and artificial intelligence (AI) will generate more realistic and comprehensive models. Better models will more efficiently capture the complexity of metastasis and incorporate various factors such as genetic mutations, cellular interactions, and microenvironmental influences.

Predictably, the future of cancer metastasis models will likely be tailored to individual patients by integrating patient-specific data, such as genomic information, molecular profiles, and clinical data, models. Metastasis models will be highlighted by integrating data from multiple omics technologies, such as genomics, transcriptomics, proteomics, and metabolomics. The combination of these diverse datasets enhances a more comprehensive understanding of the molecular mechanisms underlying metastasis in identifying novel biomarkers, therapeutic targets, and potential drug combinations (139).

Future models will also focus on accurately exploring the interactions between cancer cells and their surrounding microenvironment, including immune cells, stromal cells, and extracellular matrix components. This holistic approach will be applied to the dynamic processes driving metastasis and facilitate the development of targeted therapies.

Combining *in vitro* and *in vivo* systems will likely be crucial in studying cancer metastasis despite that in silico models are valuable. *In vitro* models, such as organoids and 3D cultures, can capture specific aspects of cancer biology, while animal models offer a more systemic view. Integrating these approaches will enhance our understanding of metastasis and enable the evaluation of therapeutic interventions.

Cancer metastasis models will continue to play a vital role in high-throughput screening platforms for drug discovery. Utilizing large-scale screening approaches benefits researchers in identifying potential drugs specifically targeting metastatic processes. These models can also be used to predict drug response and optimize treatment regimens for individual patients (140).

Collaboration and data sharing among researchers and institutions will be crucial for advancing cancer metastasis models. Integrating diverse datasets from various research groups will enable the development of more robust and generalizable models. Initiatives like data consortia and open-access repositories will facilitate the sharing of data, methods, and models, accelerating progress in the field (141).

**Figure 7:**
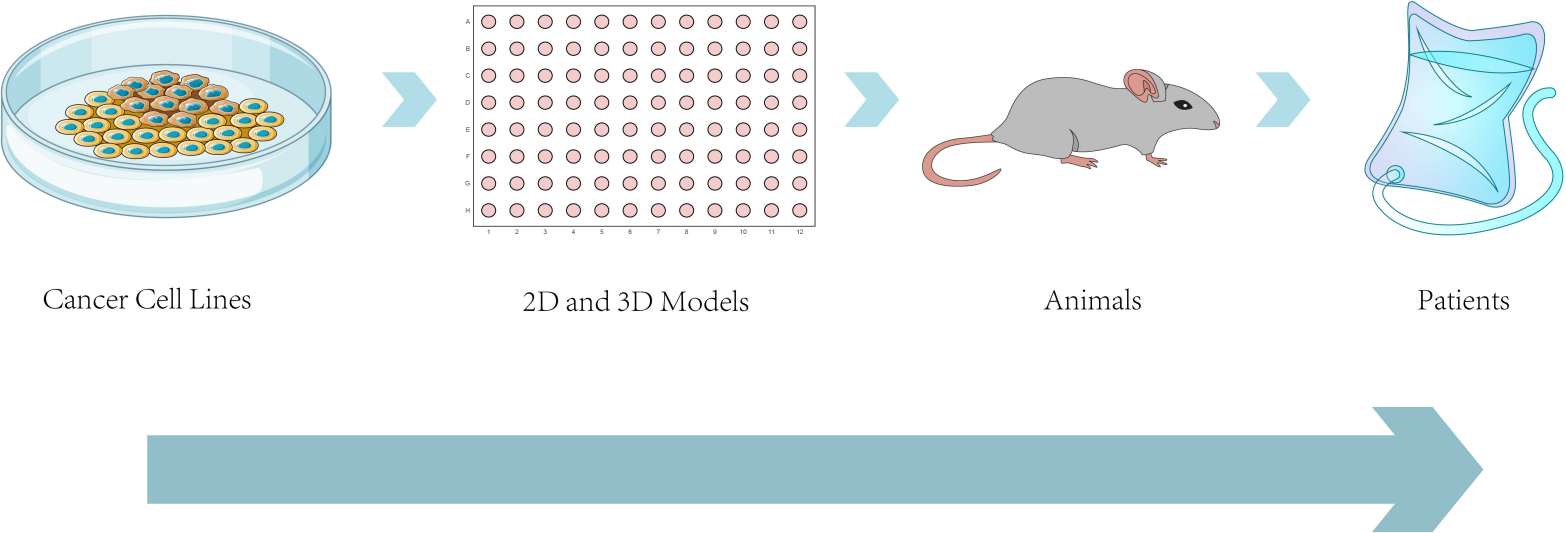
Approaches to modeling cancer metastasis: from bench to bedside. This schematic illustrates the progressive modeling systems used to study cancer metastasis, from *in vitro* cancer cell lines and 2D/3D cultures (e.g., organoids, spheroids) to *in vivo* animal models (e.g., PDX, GEMMs),ultimately aiming at clinical applications in patients. The diagram emphasizes the increasing biological complexity and translational relevance across model systems, demonstrating the manner preclinical platforms underlie the development of patient-tailored therapies and inform clinical decision-making for metastatic cancer.

The future of cancer metastasis models is promising. Advances in computational modeling, personalized medicine, multi-omics integration, microenvironment modeling, hybrid *in vitro*/*in vivo* approaches, high-throughput screening, and collaborative efforts will revolutionize our understanding of metastasis and aid in developing effective treatments for cancer patients(**Figure 7**).
